# Differential Nuclear and Mitochondrial DNA Preservation in Post-Mortem Teeth with Implications for Forensic and Ancient DNA Studies

**DOI:** 10.1371/journal.pone.0126935

**Published:** 2015-05-19

**Authors:** Denice Higgins, Adam B. Rohrlach, John Kaidonis, Grant Townsend, Jeremy J. Austin

**Affiliations:** 1 Australian Centre for Ancient DNA, School of Earth and Environmental Sciences and Environment Institute, University of Adelaide, South Australia, 5005, Australia; 2 School of Mathematical Sciences, University of Adelaide, South Australia, 5005, Australia; 3 School of Dentistry, University of Adelaide, South Australia, 5005, Australia; University College Dublin, IRELAND

## Abstract

Major advances in genetic analysis of skeletal remains have been made over the last decade, primarily due to improvements in post-DNA-extraction techniques. Despite this, a key challenge for DNA analysis of skeletal remains is the limited yield of DNA recovered from these poorly preserved samples. Enhanced DNA recovery by improved sampling and extraction techniques would allow further advancements. However, little is known about the post-mortem kinetics of DNA degradation and whether the rate of degradation varies between nuclear and mitochondrial DNA or across different skeletal tissues. This knowledge, along with information regarding ante-mortem DNA distribution within skeletal elements, would inform sampling protocols facilitating development of improved extraction processes. Here we present a combined genetic and histological examination of DNA content and rates of DNA degradation in the different tooth tissues of 150 human molars over short-medium post-mortem intervals. DNA was extracted from coronal dentine, root dentine, cementum and pulp of 114 teeth via a silica column method and the remaining 36 teeth were examined histologically. Real time quantification assays based on two nuclear DNA fragments (67 bp and 156 bp) and one mitochondrial DNA fragment (77 bp) showed nuclear and mitochondrial DNA degraded exponentially, but at different rates, depending on post-mortem interval and soil temperature. In contrast to previous studies, we identified differential survival of nuclear and mtDNA in different tooth tissues. Futhermore histological examination showed pulp and dentine were rapidly affected by loss of structural integrity, and pulp was completely destroyed in a relatively short time period. Conversely, cementum showed little structural change over the same time period. Finally, we confirm that targeted sampling of cementum from teeth buried for up to 16 months can provide a reliable source of nuclear DNA for STR-based genotyping using standard extraction methods, without the need for specialised equipment or large-volume demineralisation steps.

## Introduction

Advances in DNA analysis of human skeletal remains are providing high-resolution insights into the origin [1], migrations [2], health [3], biogeographic ancestry [4,5], phenotype [6,7] and identification [8–10] of deceased individuals and populations for evolutionary, archaeological, medical and forensic studies. Much of this progress has resulted from post-DNA-extraction advances in polymerase chain reaction (PCR) sensitivity [11], the design and optimization of short-amplicon DNA typing technologies [4], and next-generation sequencing [1,2] that focus on the small amounts of highly degraded DNA recovered from skeletal remains. In contrast, sampling and DNA extraction techniques from bones and teeth have remained largely unchanged since the earliest publications in ancient DNA and forensic biology [12,13] over two decades ago. These first steps in DNA analysis of skeletal remains are critical and can have a major impact on the amount and integrity of recovered endogenous DNA [14–17], contamination [18], and co-extraction of PCR inhibitors [13,19,20], thereby dramatically affecting the success of downstream analysis.

While the differential preservation of DNA in various skeletal elements has been considered [21,22], relatively little attention has been paid to identifying those skeletal tissues with high ante-mortem DNA content or the relative rates of post-mortem DNA degradation within and between different skeletal tissues. Resolving these issues is critical to future improvements in DNA analysis of skeletal remains and could clarify intra- and inter-individual variation in DNA content of skeletal tissues leading to better predictive models for sample selection. The precise selection of tissue for post-mortem sampling is not only crucial to maximise recovery of endogenous DNA but also to minimise destructive sampling, the potential for contamination, the co-extraction of inhibitors and the need to remove large amounts of inorganic (hydroxyapatite) and organic (collagen) fractions. These non-DNA components of bone and teeth are primarily responsible for the large volume/low throughput/high cost nature of DNA extractions from skeletal remains [16,23], and represent potent PCR inhibitors, if not completely removed during the extraction process [24]. Informed targeted sampling would also allow for smaller sample sizes to be processed facilitating the use of medium- to high-throughput techniques and standard laboratory equipment, leading to considerable cost and time saving.

Post-mortem DNA damage has been well characterised, based on theoretical and *in-vitro* studies, and empirical observations of DNA recovered from ancient and degraded samples [25–27]. However, little is known about the kinetics of post-mortem DNA degradation in a real world situation, nor how this varies across different tissues and skeletal elements. Environmental conditions (e.g. temperature, moisture and pH) in combination with time since death (post-mortem interval-PMI) are thought to be the primary factors influencing DNA degradation but the relative effects of environment and time appear to be strongly situation dependent, leading to claims that the rate of DNA degradation cannot be predicted. In contrast, recent work suggests that under a range of conditions, DNA degradation follows a random fragmentation model [28,29] and, at least in bone, that the rate of mitochondrial DNA (mtDNA) degradation can be predicted based solely on PMI and ambient temperature [28]. Whilst environmental conditions are believed to have a strong effect on DNA preservation over long PMIs, it is uncertain whether these factors are important over shorter time spans. It also appears that, over long PMIs (hundreds to thousands of years), mtDNA degrades at a slower rate than nuclear DNA [28,30]. It is not known however if the rate of DNA degradation varies across different skeletal elements (bone types and tooth tissues), nor whether the long-term degradation rate and differential degradation of mtDNA vs. nuclear DNA applies at forensically relevant timescales (days-years). Resolution of these issues is important as sample selection is frequently based on subjective visual interpretation of morphological preservation. In addition, the primary focus, at least for forensic identification, is on individualisation via nuclear short tandem repeats (STRs), which requires relatively large amounts of intact DNA.

Teeth, which are commonly used for forensic identifications and ancient DNA studies, offer great potential for targeted sampling at various PMIs, and to examine short-term DNA degradation. Their anatomical location (within bony sockets) and morphological structure (particularly covering of impenetrable enamel over the crown) provides unique protection to endogenous DNA from post-mortem degradation [18,31–33]. Furthermore, the contrasting cellularity and mineral content of the four tooth tissues (enamel, dentine, pulp and cementum) [34] creates a unique biochemical and anatomical setting to examine the content and post-mortem degradation of DNA. Conventional sampling of teeth for DNA analysis generally follows one of two pathways. Either targeted sampling of the pulp (the DNA-rich soft tissue component of the tooth) by splitting open the tooth or drilling in through the crown [35,36], or non-targeted sampling of the entire tooth (or root). Grinding of the entire tooth provides access to the largest amount of DNA but also includes a large amount of mineral (cementum is 45%, dentine 70% and enamel 97% composed of mineral) that must be removed prior to downstream analysis.

The objective of this study was to investigate the DNA distribution and rates of DNA degradation in the different dental tissues over short to medium post-mortem intervals. This was achieved using quantitative real time PCR (qPCR) to measure the relative degradation rates across tissues and between nuclear and mitochondrial DNA. A concurrent histological examination was also undertaken to improve understanding of the effects of post-mortem decomposition on the tooth tissues and correlate these changes with the DNA results. A high level of sample homogeneity and minimal variation in environment was achieved to gain a better understanding of the effects of ante-mortem factors, temperature and PMI on DNA content and degradation.

## Materials and Methods

### Sample collection and post-mortem decomposition

The Research Ethics and Compliance Committee of The University of Adelaide approved this research project including the consent procedure (Approval number: H-134-2009). Written informed consent was obtained from all participants and in the case of minors from a parent/guardian. A total of 150 third molar teeth, free from dental disease, were collected from 85 donors along with a blood sample for reference profiles, all samples were subsequently de-identified. Only third molar teeth were used, both to reduce variables and because these teeth were available in large quantities from a range of age groups. Donor age varied from 16 to 60 years, with a male to female ratio of 38:47. Teeth were randomly allocated to one of six groups (25 teeth per group: 19 for DNA analysis and six for histological examination). No individual contributed more than one tooth to each group. The six groups represented six PMIs (zero months, one month, two months, four months, eight months and 16 months). To visualise post-mortem structural change over an extended time period two teeth (one incisor and one molar) known to be over 500 years PMI were also studied histologically but were not included in data analysis.

All teeth, other than those in the zero month PMI group, were buried approximately 20 cm deep, randomly spread across two galvanized steel raised beds containing sandy loam. The beds measured 1 m high x 1 m wide x 3 m long and were situated outside without protection from the weather. The beds were located approximately 50 km southeast of Adelaide, South Australia. Mean maximum and minimum temperatures vary from 27.4–13.6°C in summer (January) to 14.8–5.9°C in winter (July). Rainfall (mean annual = 491 mm) is biased towards winter (64mm in July compared to 19.8mm in January). To facilitate sample retrieval a wire grid was placed on the surface of each bed allowing a grid reference to be recorded against each tooth. Temperature readings were taken at regular intervals (several times a month for short burials and once a month for longer term burials) from six sites (one at each end and one in the middle of each bed, numbered 1–3 in box 1 and 4–6 in box 2). A burial temperature for each tooth was determined by calculating the average of the temperature recordings from the site closest to the tooth over the course of its PMI.

At the end of each PMI the teeth were retrieved, using stringent collection protocols to minimise risk of contamination, including the use of fresh gloves for each tooth and wiping of all excavation equipment with 3% sodium hypochlorite between retrieval of each tooth. Once a tooth was retrieved it was freed from the bulk of soil by gentle rubbing between gloved fingers and placed in an individually numbered sterile container.

### Histology

Teeth for histological examination were briefly rinsed under running water to remove blood or dirt then immersed in 10% neutral buffered formaldehyde for 72 hours. Subsequently the teeth were rinsed overnight under running tap water then incubated at room temperature in 10% Ethylenediaminetetraacetic acid (EDTA), pH 7.4, with constant stirring until totally demineralised (confirmed by radiographic analysis). After demineralisation, teeth were sectioned into 1/3 and 2/3 sections in a vertical plane, embedded in paraffin wax and sliced in 7 μm sections (starting from the cut sides), slide mounted and stained with Mayer Lillie haematoxylin and counter stained with 1% eosin with phloxine. Haematoxylin allows visualisation of nuclear material as it binds to the chromatin in the DNA/histone complex, staining it a dark violet colour.

### DNA sampling and analysis

Prior to sampling, teeth for DNA analysis were carefully cleaned of blood/soft tissues or soil with DNA free water and allowed to dry. Cementum samples, in the form of a coarse powder, were scraped from each tooth using a new disposable scalpel blade for each sample. Cementum was identified visually, with sampling restricted to prevent accidental inclusion of dentine. Subsequently the crown was removed from each tooth by cutting a notch, with a diamond disc, at the cementum-enamel junction to a depth of 1mm before striking with a hammer and chisel. Any residual pulp tissue was collected, and then samples were taken of coronal dentine and then root dentine. Dentine samples were generated using hand turned wire drills and a triangular shaped hobby tool blade, with fresh tools for each sample. Not all dentine was collected to avoid inclusion of cementum or enamel in the sample and to be of similar mass to cementum samples. All equipment and workbenches were cleaned with 4% sodium hypochlorite before and after sampling each tooth. Samples were weighed after collection to allow results to be directly compared.

### DNA extraction

All pre-PCR work, including tooth sampling, was performed in a dedicated laboratory located in a separate building to the post-PCR laboratory, following strict protocols including the use of appropriate personal protective equipment. DNA extractions were performed using 14.8–81.5 mg of powdered dentine/cementum using the QIAmp DNA Investigator kit (QIAGEN, Hilden, Germany), following the manufacturer’s instructions for bones and teeth, including the use of poly-A carrier RNA. Reference samples (blood on sterile gauze) were extracted in the same fashion but on a separate day. Samples were eluted in a final volume of 60 μL. One extraction blank for every three teeth (nine samples) was included in each set of extractions. Extracts were stored at -20°C until quantification and STR profiling.

Total demineralisation of bone and tooth samples has been shown to improve DNA yields from ancient and degraded samples [16]. Hence, for teeth in the 16-month decomposition period, tissues available in sufficient quantity were divided into two samples to allow examination of the benefits of decalcification prior to extraction. Twelve of the 19 16-month teeth had sufficient tissue mass to allow dual sampling and extraction. This provided 35 paired samples, 12 from cementum, 11 from coronal dentine and 12 from root dentine. The divided samples weighed between 21.3–70.2 mg. Where two paired samples were collected one was subjected to the standard extraction process (above). The second sample was demineralised and digested overnight in 1 mL of 0.5 M EDTA, 0.5% sodium dodecyl sulfate (SDS) and 0.2 mg/mL Proteinase K at 56°C on a rotary mixer. On the following day these samples were centrifuged to pellet undigested material then the supernatant was transferred to an Amicon Ultra-4 centrifugal filter (Millipore) and centrifuged at 4000 xg for 10 min. Subsequently 1mL DNA free water was added and the sample was again centrifuged for 5 min repeatedly until the residual volume was equal to or less than 200 μL. Once the desired volume was reached 280 μL of ATL was added and the resultant supernatant was transferred to a 2 mL tube and treated in the same manner as the non-demineralised samples for DNA extraction and downstream analysis.

### DNA Quantification

Quantification of DNA was performed using real time quantitative PCR (qPCR) with SYBR green chemistry. DNA was quantified using three, previously published, primer sets. Cycling conditions included a 5-minute denaturation step at 95° C, 45 cycles of 95° C for 10 seconds, 58° C for 20 seconds, and 72° C for 15 seconds (for the mitochondrial primers the annealing temp was increased slightly to 59°). The specificity of primers to a single binding site was assessed using a post qPCR melt curve to visualize the dissociation kinetics. Primer details and references are shown in Table 1.

**Table 1:** Details of primers used for quantification of DNA.

Fragment length and reference	Primer name	Primer Sequence 5’-3’
mtDNA 77 bp [14]	L13258/H13295	ATCGTAGCCTTCTCCACTTCAA AGGAATGCTAGGTGTGGTTGGT
Nuclear DNA 67 bp [37]	HomoSap_CSF STR_F/ HomoSap_CSF STR_R	GGGCAGTGTTCCAACCTGAG GAAAACTGAGACACAGGGTGGTTA
Nuclear DNA 156 bp [38]	HomoSap DQARB1_105F/ HomoSap DQARB1_214R	AGGTTGCTAACTATGAAACACTGGC TGGTTTAGGAGGGTTGCTTCC

The qPCR mix consisted of 5 μL 2x Brilliant II SYBR green master mix (Agilent Technologies, USA), 0.15 μM forward primer, 0.15 μM reverse primer, 400 ng/μL Rabbit Serum Albumin, 3.3 μL water and 1 μL DNA extract to a total of 10 μL. All samples were run in triplicate and negative (PCR blank) and positive controls (dilutions of male genomic control DNA, Applied Biosystems, USA) were included on all runs. Extraction blanks were also quantified. Real time PCR was performed on a Corbett 6000 Rotorgene thermocycler.

DNA concentration was determined using the comparative C_T_ method; unknown samples were compared to a standard curve using the Rotor-Gene 600 Series Software 1.7. The arithmetic mean value of the triplicate qPCR results was calculated for each sample for inclusion in the final analysis.

### STR genotyping

Amplification was carried out using Profiler Plus (Applied Biosystems, USA). Cycling was performed on a 9700 GeneAmp cycler and consisted of an initial denaturation at 95°C for 10 min followed by 28 cycles of 94°C for 1 min, 59°C for 1 min and 72°C for 1 min, followed by a final extension at 60°C for 45 min. All reactions were initially performed in 25 μL volumes, with 10 μL of DNA (diluted when appropriate to allow a final concentration of ideally between 0.5 and 1 ng). Capillary electrophoresis was performed using an Applied Biosystems 3130xl genetic analyser and profiling analysis was undertaken using GeneMapper ID v3.2.1. The analysis was repeated in duplicate or triplicate, in 12.5 μL reaction volumes including 5 μL of DNA, when peak heights were lower than 50 Relative Fluroscence Units (RFU). A consensus approach was then used to identify peaks from the multiple reactions

### Statistical analysis

In order to standardise for different sample weights DNA yields from cementum, coronal and root dentine were converted to ng of DNA per mg tooth tissue (ng/mg) for nuclear DNA, and fragment copies per mg of tooth tissue (copies/mg) for mtDNA. As pulp was sampled as a whole, it was not weighed and is thus reported in ng of DNA per μL of extract (ng/μL). A probability level of < 0.05 was considered significant for all statistical tests.

A regression analysis was conducted for each of the three quantified fragments (nuclear 67 bp, nuclear 156 bp and mt 77 bp) using the chronological age of the donor (in years), the sex of the donor, the plot the tooth was buried in, the PMI time (in months), the (arithmetic) average soil temperature of the burial plot over the period of interment, and tissue type as regression predictor variables to determine relationships. A complete model with interaction terms up to order five was fitted. Examination of the residuals indicated non-linearity. The Box-Cox transformation of the response variable (DNA concentration) was applied using the *boxcox* function from the MASS library in R. This indicated a log transform of the response variable was appropriate.

A linear mixed effects model was fitted using the R-package ‘lme4’ [39], and for each fragment type, a backwards step procedure using the significance of predictors was applied to the full interaction model. For simplified models, an analysis of variance test was used to determine if the model had been significantly modified [40].

To more closely examine the effects of chronological age on DNA yield for each type of DNA fragment, the data were further subset into two groups of teeth those that were buried, and those, that were not. As before, a linear mixed effects model was fitted to each subset of the data. For the unburied teeth, the age of the subject and the tissue sampled were treated as fixed effects, and the individual was treated as a random effect. For the teeth that had been buried, the age of the subject, the decomposition time, the average temperature and the tissue sampled were treated as fixed effects, and the individual and plot were treated as random effects.

To compare the rate of decay of the three different fragments, a comparison of half-lives was made from the fitted transformed linear models, for various average temperatures.

A discussion with regards to the statistical analysis performed in this study can be found in S1 File, **Section 3 Statistical analysis—on the topic of p-values and prediction and confidence intervals for linear mixed effects models.**

## Results

The average soil temperature calculated for each burial ranged between 15.8°C and 20.5°C with a maximum difference between sites never exceeding 2°C. (See **Table A and B in S1 File** (**Section 1 Temperature Readings)** for details of instrumentation, individual readings and standard deviations)

### Histology

**Fig 1:**
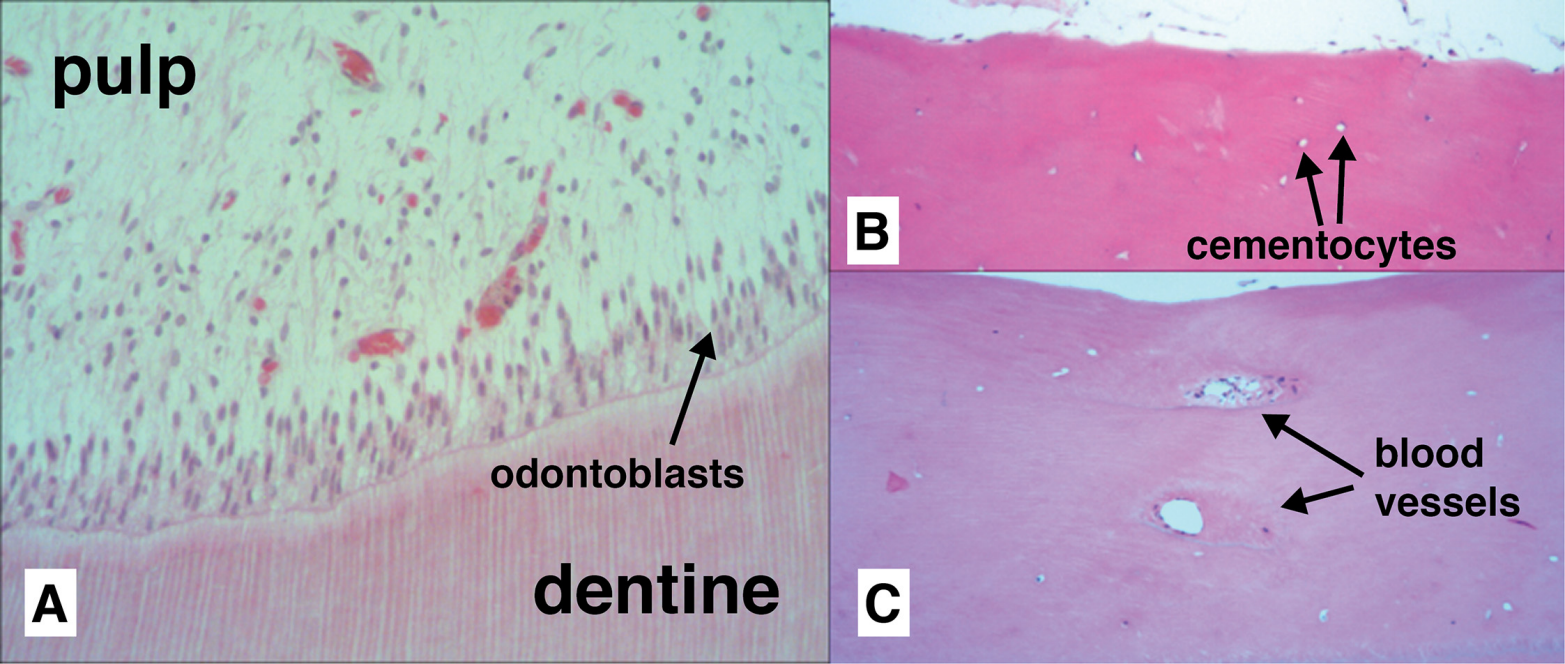
Histological appearance of fresh teeth at 200x magnification. A: Pulp tissue is rich in odontoblasts (cells that form dentine), fibroblasts, defence cells (e.g. histocytes and macrophages), plasma cells, nerve cells and undifferentiated mesenchymal cells; B: Cellular cementum containing cementocytes, in spaces comparable to osteocytic lacunae, with cementoblasts visible on the surface; C: Cementum also had soft tissue inclusions and blood vessels present.

**Fig 2:**
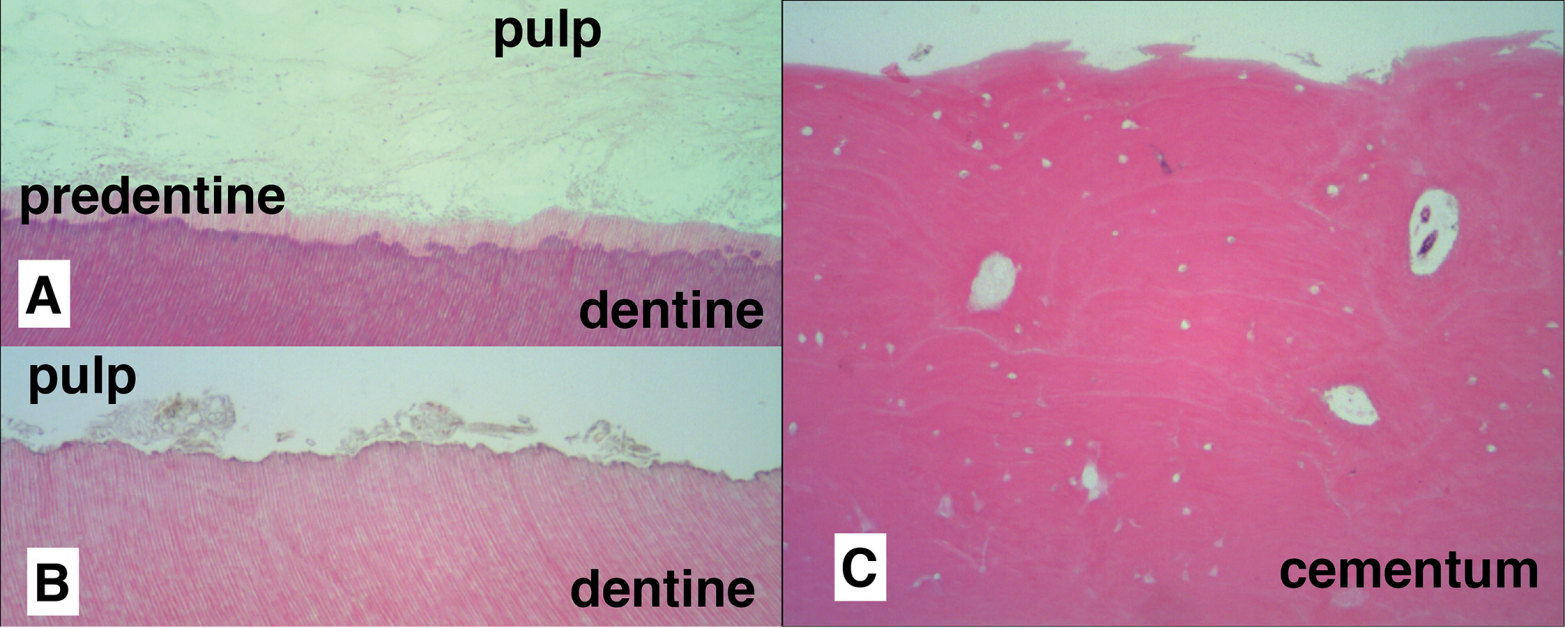
Histological appearance of buried teeth at 40x (A, B) and 200x (C) magnification after A: one month—pulp tissue shows loss of structure and nuclear material; B:16 months—very little pulp tissue remains with much of the pulp chamber being empty. Dentine displayes almost complete loss of the predentine layer; and C: 16 months—cementum shows little structural change but loss of cells in soft tissue inclusions and on the external surfaces.

**Fig 3:**
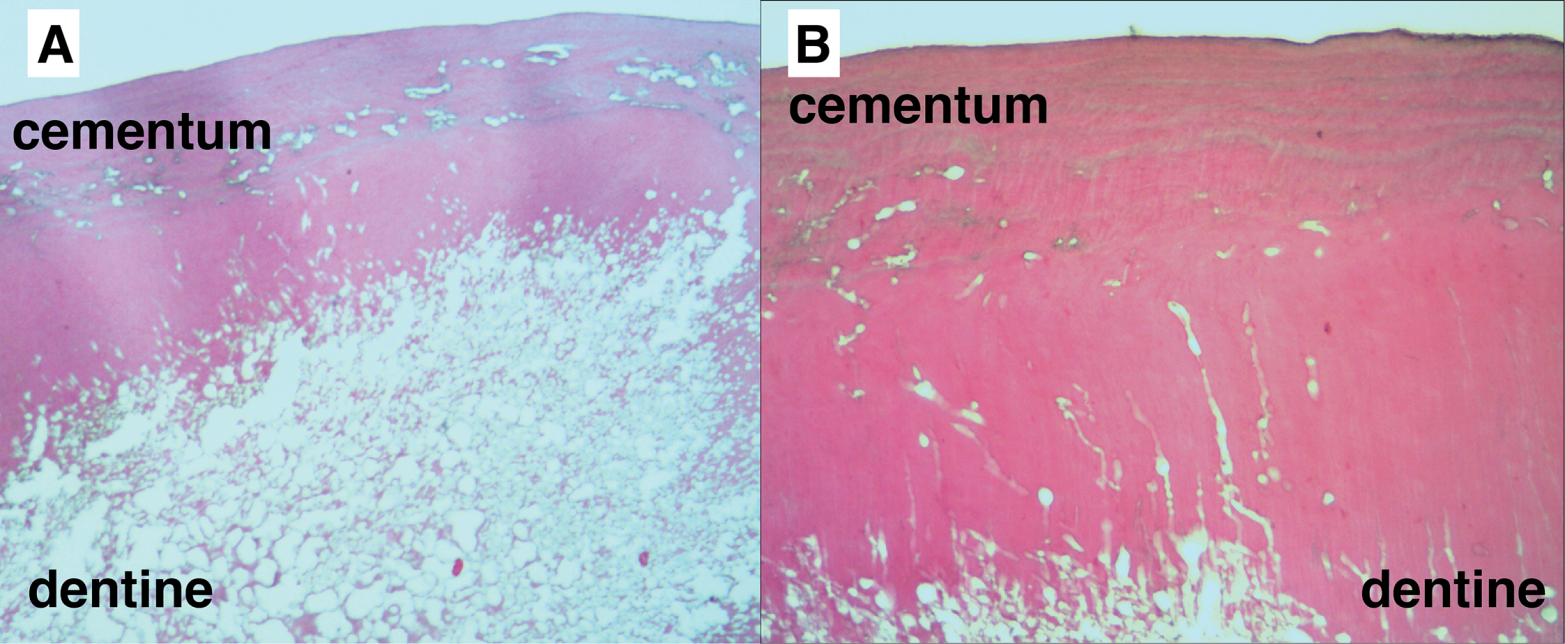
Histological appearance of teeth with a PMI >500 years at 40x (A) and 100x (B) magnification. A: Marked structural breakdown of the dentine and no visible cellular material. Areas of focal destruction in the cementum; and B: Destruction of the dentine traveling outwards along the dentinal tubules with almost the full thickness of the dentine being affected.

Non-decomposed teeth showed pulp tissue rich in nucleated cells, with nucleated cells visible in cementum and a layer of cementoblasts on the external root surfaces (Fig 1A and 1B). Nuclei were also noted in blood vessels and soft tissue inclusions within the cementum (Fig 1C). In decomposed teeth, pulp tissue showed loss of structural integrity and cellular detail by one-month post-mortem, with a large decrease in presence of nuclei in comparison to fresh teeth (Fig 2A). Teeth subjected to longer PMIs showed a further decrease in the presence of nuclei and an increasing absence of pulp tissue remnants (Fig 2B). Dentine showed structural changes in the predentine layer, with the pulpal surface becoming increasingly irregular until at 16 months PMI the predentine layer was completely lost in most cases (Fig 2B). However, over the same time period minimal structural change was seen in the cementum layer although a decrease in cementoblasts on the root surfaces and nuclei in soft tissue inclusions was noted (Fig 2C). In the ancient teeth (PMI > 500yrs) no nuclear material was visible, structural breakdown was seen extended through much of the dentine, and some focal destruction was visible in the cementum (Fig 3A and 3B). However, as in the buried teeth, the cementum was far less affected structurally than the dentine.

### DNA yields

**Fig 4:**
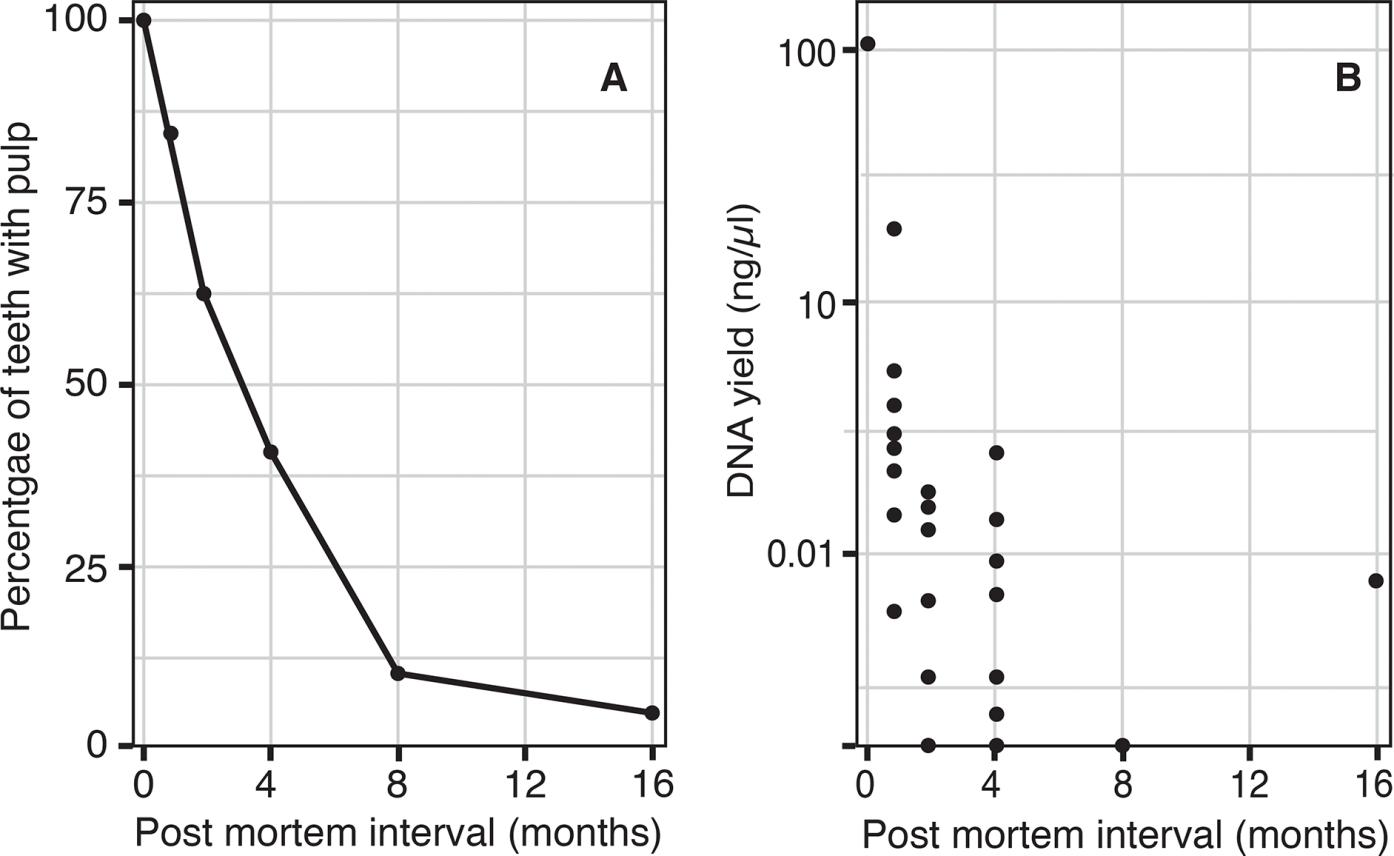
Degradation of pulp tissue in buried teeth with PMIs from 0–16 months. A: percentage of teeth at each time period that contained visible pulp, and B: yield of nuclear DNA from pulp tissue (quantified using the nuclear 67 bp fragment).

Both the percentage of teeth with visible pulp, and the yield of nuclear DNA obtained from pulp tissue when present, declined rapidly from 0–8 months PMI (Fig 4). By eight months only 10% of teeth had any remaining visible pulp tissue and only one tooth beyond four months yielded quantifiable nuclear DNA.

**Fig 5:**
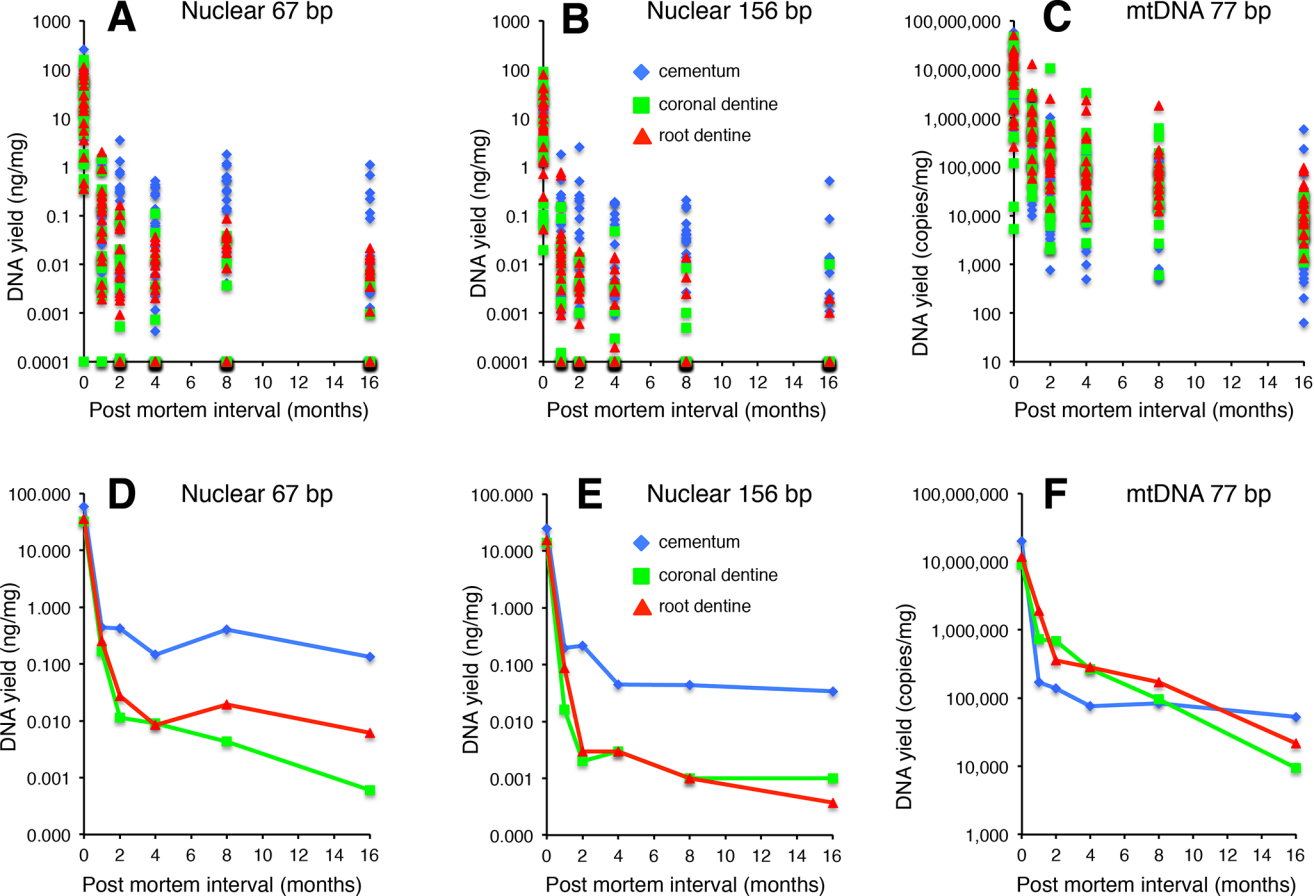
DNA yields at each PMI interval (1–16 months) for three tooth tissues (cementum, coronal dentine and root dentine) for two nuclear DNA targets (67 bp and 156 bp) and one mtDNA target (77 bp). A-C: DNA yield for each tooth, and D-F: Arithmetic mean DNA yield at each PMI interval.

The yield (taken as the average of qPCR triplicates for each sample) of two nuclear DNA fragments (67 bp and 156 bp) and one mtDNA fragment (77 bp) for cementum, coronal dentine and root dentine declined exponentially over the 16 month PMI range (Fig 5). Beyond one month PMI, average nuclear DNA yields from cementum were 1–2 orders of magnitude higher than either of the averages for the dentine samples. Average mtDNA yields from dentine were higher than cementum for PMIs between 1–8 months, but fell below that of cementum at 16 months PMI. Both the sex of the donor and burial plot were insignificant factors.

**Fig 6:**
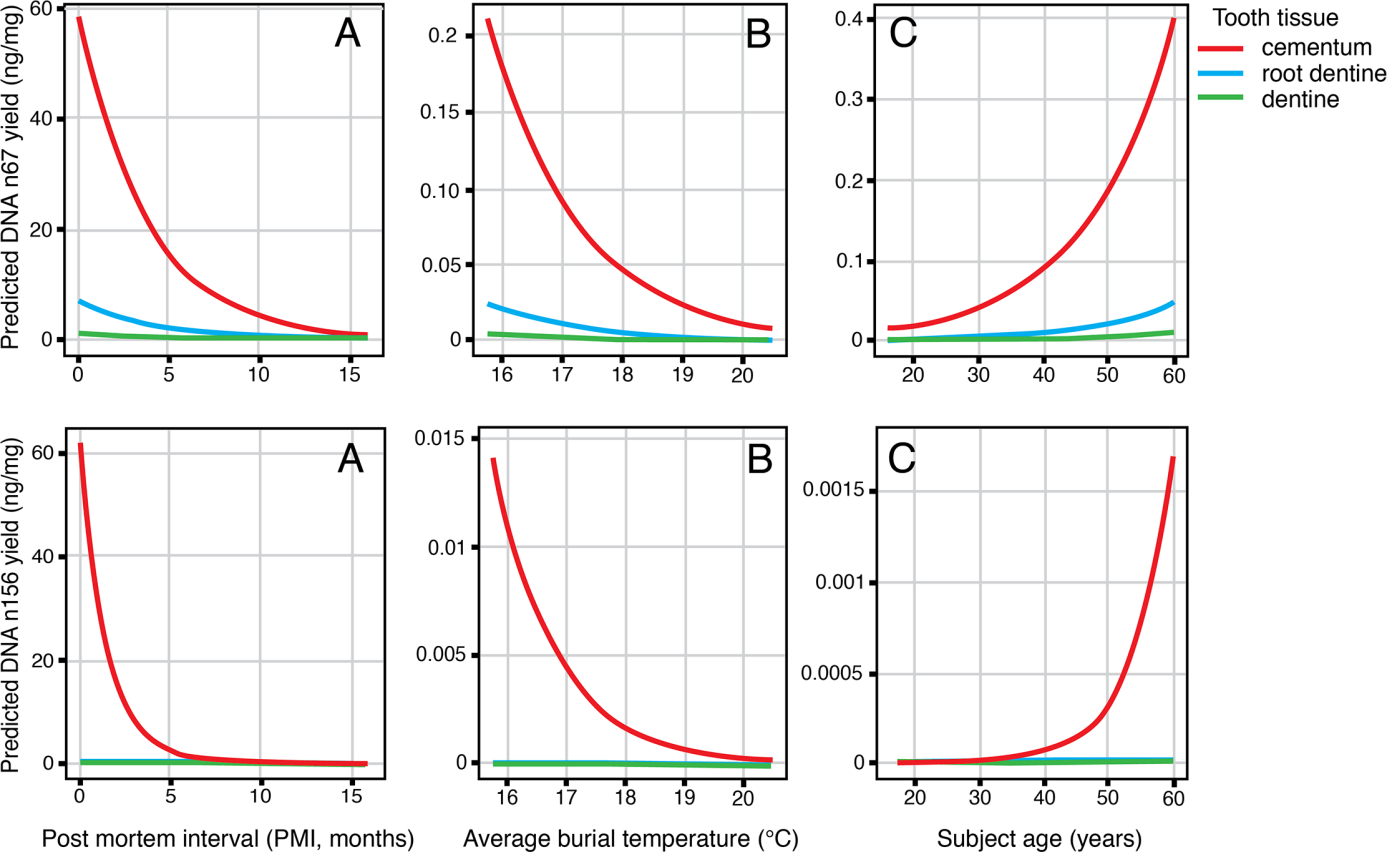
Predicted fragment yield for nuclear targets at varying PMI, soil temperature and subject age. PMI, soil temperature and subject age are held constant at sample median values where applicable. (Note the effect of PMI was much greater than that of soil temperature and age, the Y-axis scales reflect this.)

For both nuclear fragments, tissue type, PMI, average soil temperature and subject age were significant determinants of DNA yield (Fig 6). For the nuclear 67 bp fragment, interaction terms between PMI and average temperature, and PMI and subject age were also significant (p-value = 0.0215). For the nuclear 156 bp fragment, the interaction term between PMI and average soil temperature was significant (p-value = 0.03034). For the nuclear fragments, since there was significant interaction between chronological age and PMI, the half-life for a subject of median age was produced.

For all observed values of the predictor variables, predicted nuclear DNA yield from cementum was significantly higher than from dentine. Longer PMI times and higher average burial temperature had a negative effect on nuclear DNA yield. Conversely, an increase in subject age had a positive effect on nuclear DNA yield.

**Fig 7:**
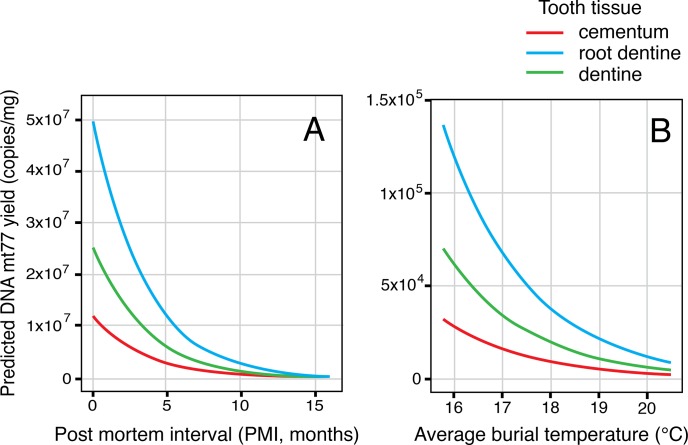
Predicted fragment yield for the mtDNA 77 bp fragment at varying PMI and soil temperature. PMI and soil temperature are held constant at sample median values where applicable.

For the mtDNA 77 bp fragment, tissue, average temperature and PMI time were significant determinants of DNA yield (Fig 7). Age of the donor was insignificant. The interaction between PMI time and average temperature was the only significant interaction term found (p-value = 6.282x10^-4^).

For all observed values of the predictor variables, mtDNA yield from cementum was significantly lower than that from dentine, with root dentine yielding the highest amount of mtDNA. An increase in PMI time and average soil temperature had a negative effect on mtDNA yield.

**Fig 8:**
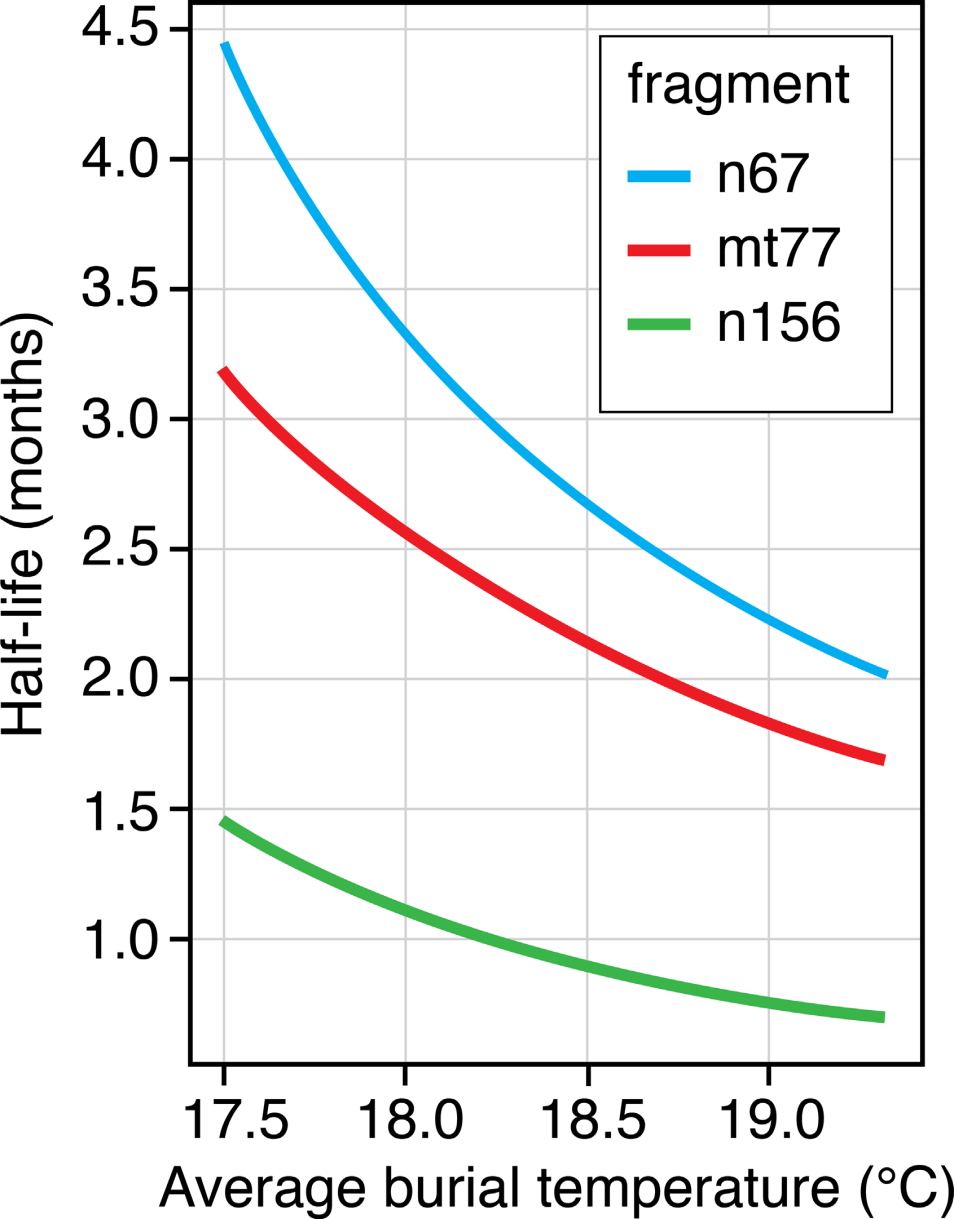
Predicted DNA half-life for varying average soil temperature for each fragment type. For nuclear DNA as the half-life is affected by donor age the average donor age has been selected for the calculation.

DNA yields for all three targets best fitted an exponential relationship, so regression equations were fitted to indicate the rate of DNA degradation in all tissue types combined (Fig 8). Soil temperature had a strong effect on the rate of DNA degradation, with as little as a 2°C rise in average temperature reducing the half-life of all fragments by two. The predicted half-life for the 67 bp nuclear fragment is 20–38% longer than for mtDNA 77 bp fragment and is 190–210% longer than for the 156 bp nuclear fragment.

**Fig 9:**
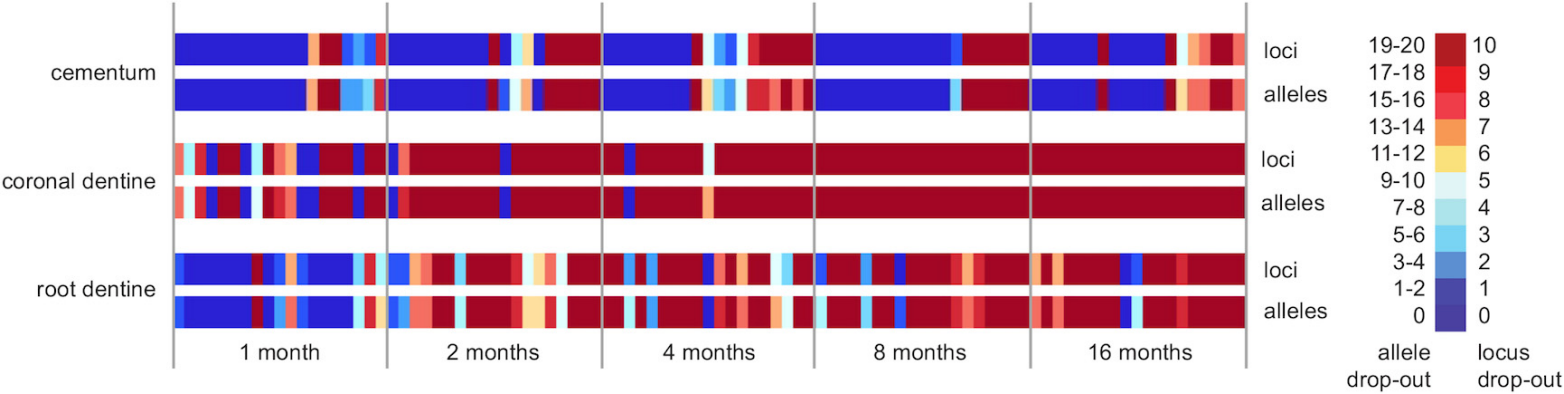
STR genotyping success for three dental tissues from five post-mortem burial intervals. Nineteen teeth were sampled for each time period (1, 2, 4, 8 and 16 months). Allele and complete locus dropout are expressed with colder colours representing lower success and warmer colours representing higher success.

STR typing outcomes achieved from cementum, coronal dentine and root dentine of the buried teeth compared to the reference profiles generated from the blood samples are displayed in the form of a heat map in Fig 9. Cementum gave the highest profiling success, with greater than 50% of samples giving full profiles at each PMI, while coronal dentine showed less than 10% full profiles after one month and root dentine less than 10% success after two months. The occurrence of dropout was seen to increase in frequency with increasing PMI. Predominantly dropout affected the longest alleles more frequently than shorter alleles.

Demineralisation of tooth powder prior to DNA extraction led to an increase in DNA yield for all samples that had DNA at detectable levels in their non-demineralised extract except in one instance. (Results are shown in **Table C** in **S1 File, Section 2 Demineralisation results**)

## Discussion

Our results demonstrate differential distribution and variable postmortem preservation of nuclear and mitochondrial DNA across within individual tissues of human teeth suggesting that targeted sampling of specific tooth tissues will improve downstream analyses benefiting in particular forensic DNA applications. Histological and qPCR analyses of teeth decomposed over short to medium postmortem intervals reveal that the structural integrity and nuclear DNA content of pulp and dentine decline rapidly, whereas cementum resists structural decay and maintains the most consistent preservation of nuclear DNA. In contrast, mtDNA is better preserved in dentine especially within the root, making the roots of teeth of more value than the coronal tissues.

More specifically, while in non-decomposed teeth (antemortem) all tissues examined yielded sufficient quantities of nuclear and mitochondrial DNA for genetic analysis, differential DNA preservation is seen across tissues of decomposed teeth (postmortem). DNA yield from all tissues declined markedly in the first month postmortem likely due to the loss of soft tissue components such as the cementoblast layer (on the surface of the roots), pulp tissue and blood vessels. This hypothesis is supported by the histological analysis, which revealed rapid breakdown of these soft tissue components. The rapid break down and loss of pulp tissue in the postmortem teeth observed in this study is in contrast to other studies where pulp has been found to persist for longer periods [35]. Other studies have reported better pulp preservation in casework samples when compared to experimental samples [31,41]. This variation in pulp degradation may relate to the post-mortem environment—with isolated buried teeth losing pulp faster than teeth that remain protected within the jawbones. With regards to the hard tissues nuclear DNA yields from dentine were reduced to very low levels in most instances by the 8-month interval, whereas nuclear DNA yields were maintained at much higher levels in cementum. This is not unexpected given the source of nuclear DNA in both tissues: the pulp for dentine, and the cementocytes for the cementum. The DNA contained in cementocytes is likely to be better protected by the mineral matrix encapsulating them.

Histology revealed structural breakdown of the dentine occurring from the pulp interface outwards. The predentine layer, which is the unmineralised layer of dentine adjacent to the odontoblast cell bodies was the worst affected. By 16 months, the predentine layer was almost completely lost with the surface of the dentine becoming irregular in appearance. In contrast, the cementum did not show obvious signs of structural change but did show loss of the external cementoblast layer and a reduction in visible nuclei in areas of soft tissue inclusions. The contrasting, preferential preservation of mtDNA in dentine may relate to the fact that dentine consists of tightly packed tubules, which contain odontoblastic cell processes, and mitochondria rich nerve fibers. During postmortem degradation the DNA within these mitochondria potentially become trapped within the dentinal tubules and are protected by the minerals of the dentine. The greater retention and extraction of mtDNA from dentine contrasts with a previous study, examining ancient tooth samples, that found that cementum was a better source of mtDNA than dentine [14]. The extensive structural breakdown of dentine seen histologically in our ancient samples suggests that the advanced structural breakdown associated with longer PMIs and loss of mineral will lead to a loss of the mtDNA contained within.

Interestingly, the chronological age of the donor had a positive effect on nuclear DNA yield from decomposed teeth. This observation may reflect an increased resistance of older teeth to decomposition. More mature teeth have narrower apices (the opening at the end of the roots) and are more heavily mineralized and less porous than younger teeth [42]. These features potentially restrict the ability of moisture and microbes to penetrate the outer layers of the tooth, reducing the effects of post-mortem decomposition and DNA degradation. The effect of age was more pronounced in cementum likely due to cellular cementum increasing in thickness with age [43]. Most probably, the cementum sampled from younger individuals was primarily acellular cementum.

The rate of degradation of mtDNA calculated in our study for buried human teeth is faster than that seen in a previous study examining ancient moa bone [28]. This is not surprising as the previous study was performed using ancient samples (minimum 1600 years old) and, as the authors noted, their results did not take into account the initial stages of post-mortem degradation, which likely occur at a more rapid rate. While very little research has been directed at DNA degradation in the short to medium term, it has been suggested that in the initial post-mortem phase the rate of DNA degradation will be more rapid, due to the activity of nucleases rather than longer term processes of hydrolysis and oxidation seen in ancient samples [29,44]. More precisely in a study by Sawyer *et al* [44] it was determined that, when examining samples with a postmortem time of less than 100 years, DNA is fragmentated to a small size in a short time after death most likely due to autolysis. Furthermore the longer half-life for nuclear 67 bp fragment over the mitochondrial 77 bp fragment observed in the present study is not expected and is contrary to previous research [28,30], although this may be as a result of fragment length and the short time span examined. In a previous study [45] the authors postulated that DNA fragmentation may be affected by base composition and hence be a function of the DNA sequence examined as well as the tissue from which it was extracted. In addition, in the present study only the rate calculated here for nuclear DNA, and not for mtDNA, was affected by chronological age and an interaction between age and PMI, which complicates comparisons between the two fragments.

Surprisingly, our results show that even relatively small variation in soil temperature has a major effect on the yield of DNA from all dental tissues. Although it is well established that environmental conditions greatly affect the rate of postmortem DNA degradation in ancient teeth samples [46], (with temperature having the greatest impact [28,46]), experimental studies examining shorter PMIs often do not note a significant effect of temperature [31,33]. Here we show soil temperature variations of as little as 2°C significantly affected the survival of both nuclear and mtDNA, indicating that storage of skeletal samples at consistent and low temperature prior to genetic analysis will minimise post-mortem and post-collection degradation of DNA.

## Conclusion

Nuclear and mitochondrial DNA is not distributed evenly throughout teeth and decays at different rates in different tissues. DNA yield from these tissues is influenced by ante-mortem (chronological age) and post-mortem factors (soil temperature and time). The rate of post-mortem degradation of DNA in teeth is dependent on post-mortem interval and soil temperature. Over short to medium, post mortem time periods even small changes in soil temperature can have a substantial impact on DNA preservation. Cementum (the mineralised outer layer of the tooth root) is particularly important for recovery of nuclear DNA as its structural integrity is maintained over extended periods, possibly providing additional protection to cellular material trapped within the mineral matrix. Therefore, small samples of dental hard tissues that are amenable to inclusion in standard laboratory workflows can produce reliable and successful STR profiling results if teeth are carefully subsampled.

## Supporting Information

S1 Fig Predicted fragment yield for nuclear 67bp fragment at varying PMI, soil temperature and subject age with prediction intervals. (TIF)

S2 Fig Predicted fragment yield for nuclear 156bp fragment at varying PMI, soil temperature and subject age with prediction intervals. (TIF)

S3 Fig Predicted fragment yield for mitochondrial 77bp fragment at varying PMI and soil temperature with prediction intervals. (TIF)

S1 File Table A. Temperature Measurements. Table B. Temperature Measurement standard deviations. Average temperature of each plot over each time frame with standard deviation. Table C. Average qPCR results for demineralisation vs no demineralization.
(DOCX)
