# Supplementary figures and images for: Differential Nuclear and Mitochondrial DNA Preservation in Post-Mortem Teeth with Implications for Forensic and Ancient DNA Studies
Source: PLoS One. 2015 May 19;10(5):e0126935. doi: 10.1371/journal.pone.0126935 (PMC4438076; doi:10.1371/journal.pone.0126935)

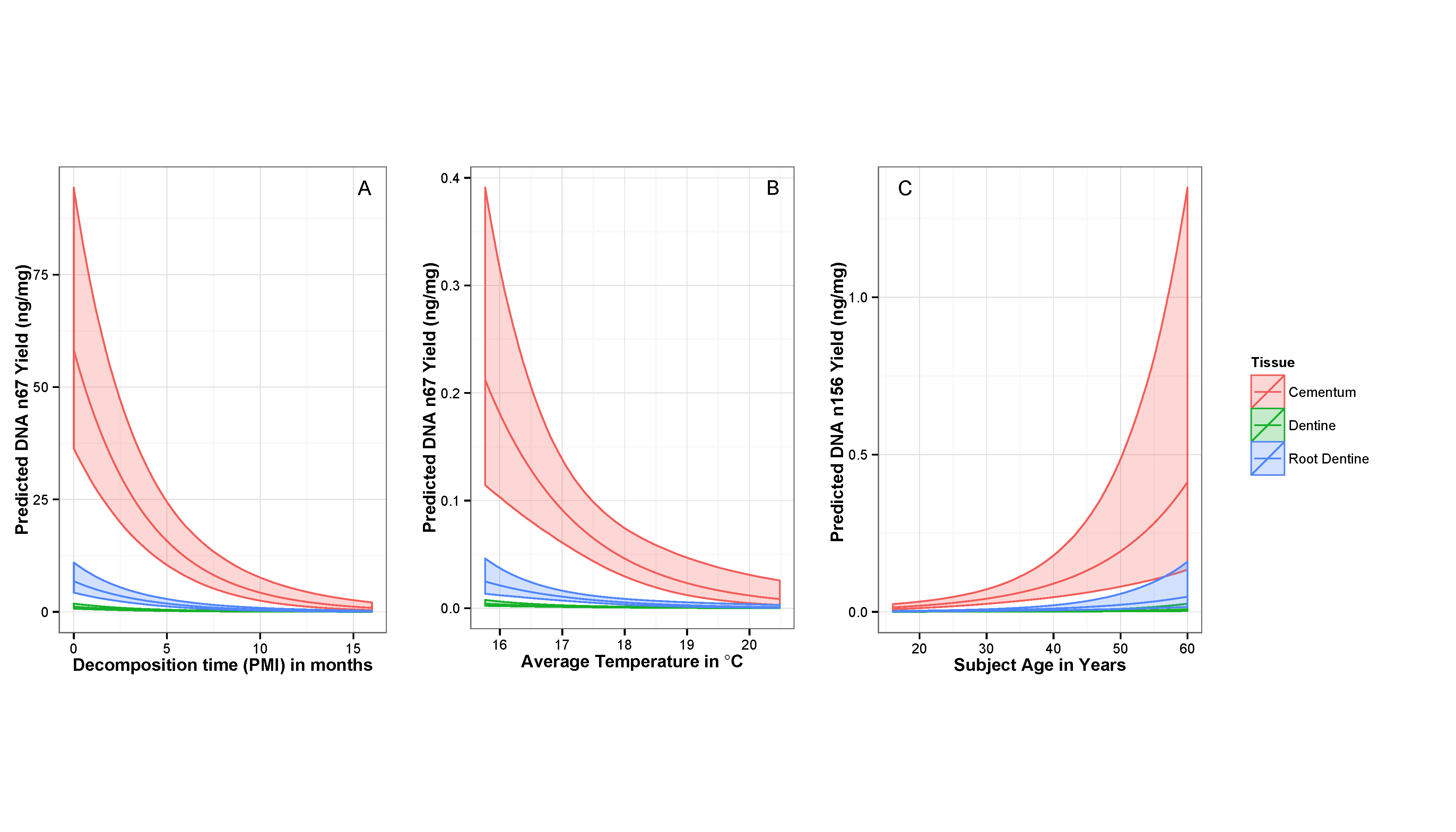

Supplement: S1 Fig — (TIF) [file pone.0126935.s001.tif]

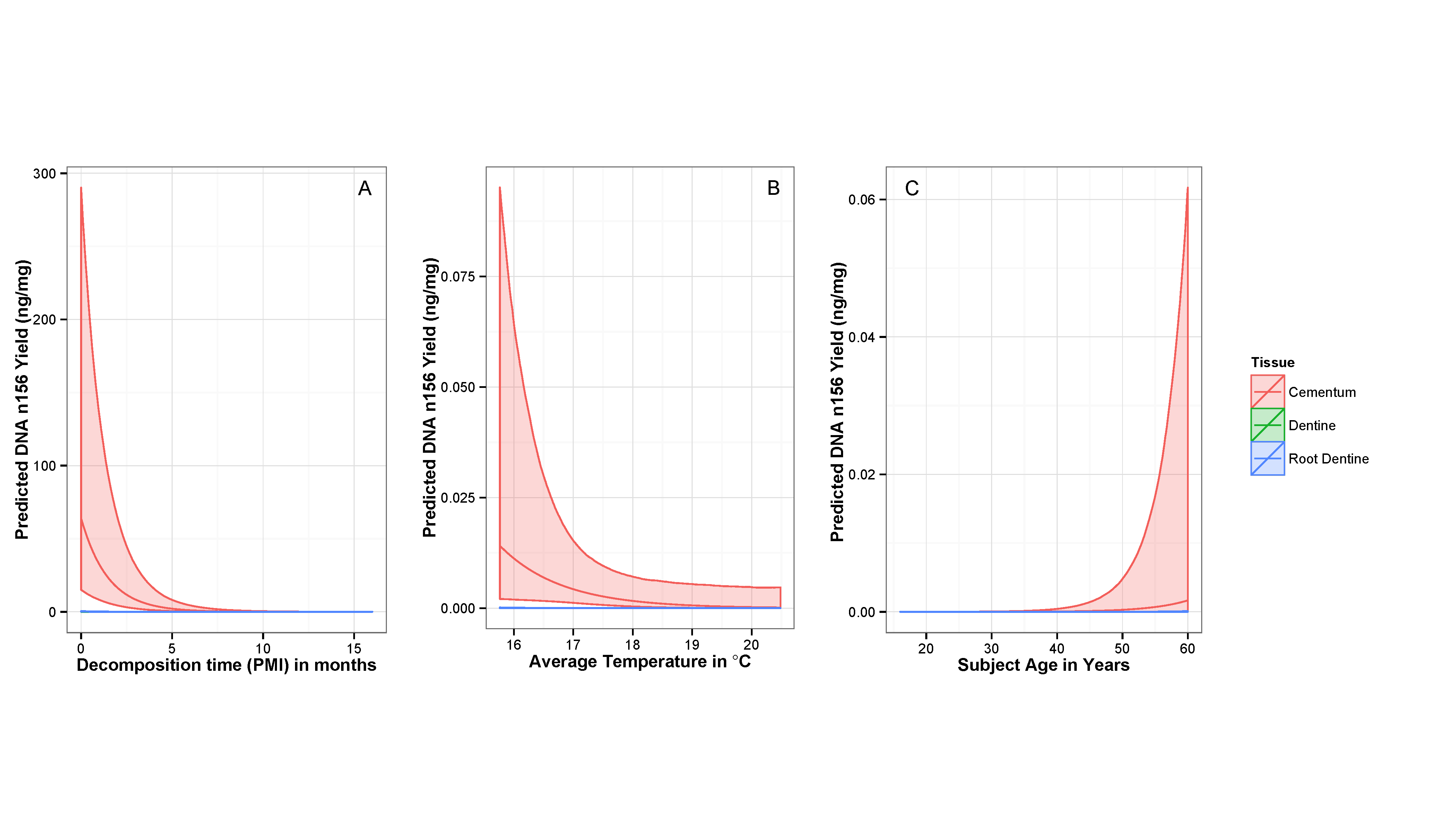

Supplement: S2 Fig — (TIF) [file pone.0126935.s002.tif]

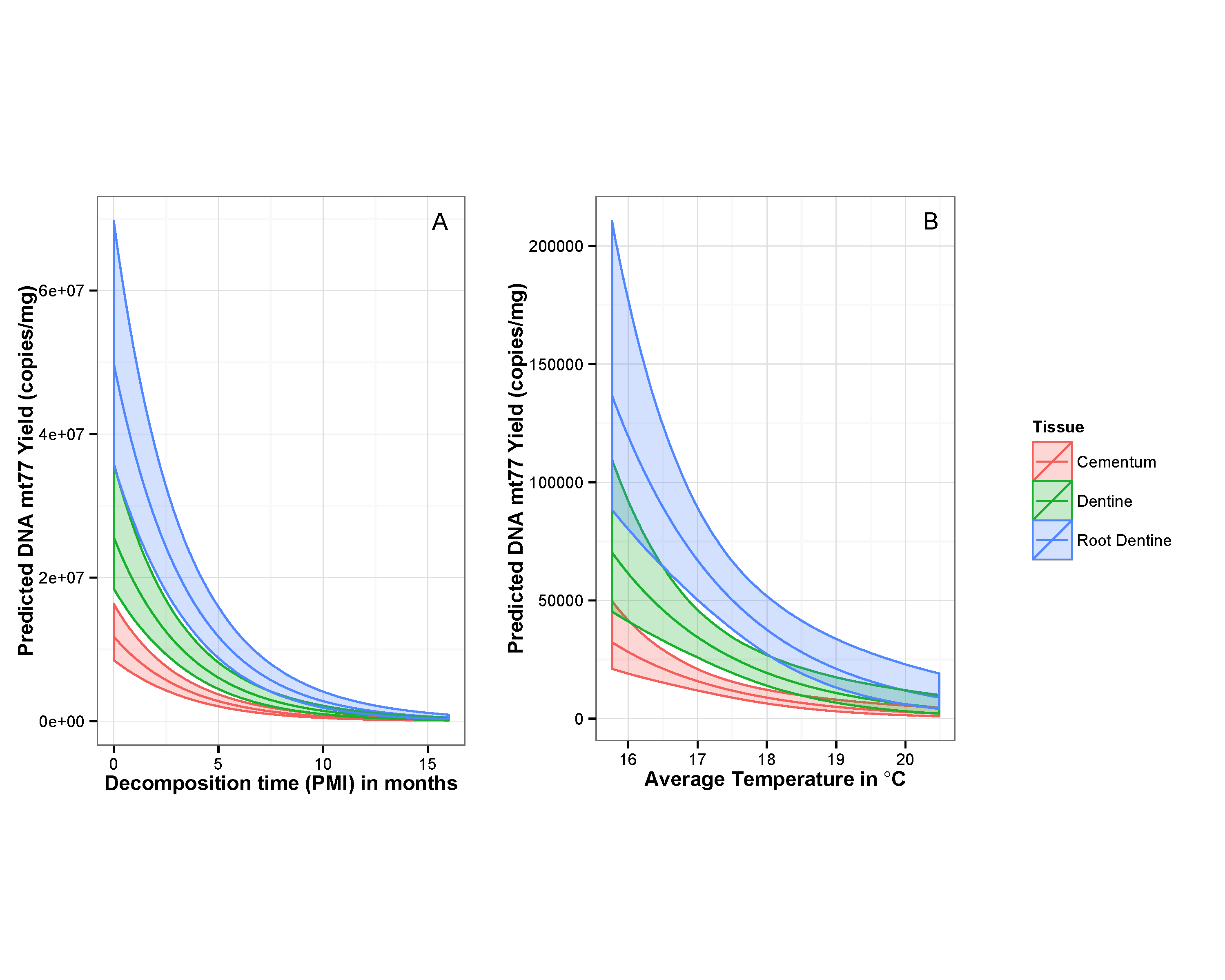

Supplement: S3 Fig — (TIF) [file pone.0126935.s003.tif]
