# Supplementary material for: Differential Nuclear and Mitochondrial DNA Preservation in Post-Mortem Teeth with Implications for Forensic and Ancient DNA Studies
Source: PLoS One. 2015 May 19;10(5):e0126935. doi: 10.1371/journal.pone.0126935 (PMC4438076; doi:10.1371/journal.pone.0126935)
Supplement: S1 File — Table B. Temperature Measurement standard deviations. Average temperature of each plot over each time frame with standard deviation. Table C. Average qPCR results for demineralisation vs no demineralization. (DOCX) [file pone.0126935.s004.docx]

# S1 File.

# Section1. Temperature readings

All soil temperature measurements were taken using a **Delta T WET Sensor** probe attached to a **HH2 Moisture Meter** (DELTA T DEVICES Cambridge England).

# Table A. Temperature Measurements

| Date | Site 1 | Site 2 | Site 3 | Site 4 | Site 5 | Site 6 |
| --- | --- | --- | --- | --- | --- | --- |
| 3/03/11 | 22.2 | NA | 22.1 | 23.3 | NA | 22.5 |
| 5/03/11 | 17.6 | 15.6 | 14.7 | 15.2 | 15.3 | 14.9 |
| 8/03/11 | 21.3 | 21.4 | 21.6 | 21.5 | 21.2 | 21.2 |
| 23/03/11 | 19.2 | 19 | 19 | NA | NA | NA |
| 5/04/11 | 20.3 | 21.8 | 21.9 | 25.1 | 25.4 | 23.1 |
| 10/04/11 | 15.7 | 15.4 | 15.5 | 16.3 | 16.6 | 16 |
| 20/04/11 | 19.1 | 18.7 | 18.9 | 19.3 | 19.6 | 19.2 |
| 26/04/11 | 19.6 | 20.4 | 21 | 22.7 | 23 | 22.4 |
| 7/05/11 | 15.9 | 15.8 | 15.9 | 16.9 | 17 | 16 |
| 16/05/11 | 14.7 | 14.8 | 15 | 15.6 | 16.2 | 16.2 |
| 29/05/11 | 13.1 | 12.6 | 12 | 13 | 12.9 | 12.5 |
| 5/06/11 | 14.6 | 13.7 | 13.3 | 15.1 | 15.6 | 14.9 |
| 9/06/11 | 11.7 | 11.4 | 11.5 | 11.4 | 11.8 | 11.4 |
| 18/06/11 | 16.9 | 15.1 | 15.3 | 17.3 | 16.6 | 16.4 |
| 23/06/11 | 14.6 | 14 | 13.9 | 14.9 | 14.5 | 14.3 |
| 26/06/11 | 14.5 | 14.3 | 15.2 | 16.7 | 15.9 | 16.1 |
| 3/07/11 | 13.8 | 13.7 | 14.1 | 14.6 | 14.3 | 15.2 |
| 20/07/11 | 11 | 10.3 | 10.2 | 11.3 | 11.1 | 10.7 |
| 1/08/11 | 15.2 | 14.7 | 15.1 | 16.5 | 16.3 | 15.6 |
| 21/08/11 | 15.3 | 15 | 15.4 | 17.3 | 17.2 | 16.1 |
| 18/09/11 | 18.5 | 20.6 | 21 | 22.9 | 23.5 | 23.1 |
| 25/09/11 | 16.8 | 15.8 | 16.1 | 16.4 | 17.1 | 16.4 |
| 9/10/11 | 15.3 | 15.8 | 15.4 | 19.8 | 16.9 | 15.5 |
| 31/10/11 | 17.9 | 19.5 | 18.8 | 21.1 | 20.5 | 18.3 |
| 27/11/11 | 29.6 | 29.2 | 31.1 | 31.5 | 29.2 | 28.5 |
| 14/12/11 | 20.4 | 20.3 | 20.4 | 21.2 | 21.4 | 20.7 |
| 3/01/12 | 30 | 30 | 32.2 | 31.3 | 31 | 30.3 |
| 18/01/12 | 24 | 25.1 | 25.5 | 26 | 26.4 | 26 |
| 13/02/12 | 20 | 21.2 | 21.3 | 22 | 22.1 | 21.8 |
| 4/03/12 | 23.7 | 22.7 | 20.5 | 21.8 | 23.3 | 24.2 |
| 1/04/12 | 17.1 | 16.6 | 16.4 | 16.5 | 17.2 | 17.4 |
| 4/05/12 | 12.1 | 11.8 | 11.6 | 11.7 | 12.2 | 12.4 |
| 6/06/12 | 10.8 | 10.6 | 10.3 | 10.4 | 10.9 | 11.1 |
| 8/07/12 | 7.7 | 7.9 | 7.7 | 8.1 | 8.2 | 7.9 |
| 22/07/12 | 14.8 | 14.9 | 15.2 | 15.9 | 15.4 | 13.9 |
| 8/08/12 | 11.8 | 11.9 | 12.1 | 12.5 | 12.2 | 11 |
| 1/09/12 | 16.6 | 15.7 | 17.3 | 20 | 20.8 | 20.2 |

# Table B. Temperature Measurement standard deviations. Average temperature of each plot over each time frame with standard deviation.

| Months Elapsed | Plot 1 | Plot 2 | Plot 3 | Plot 4 | Plot 5 | Plot 6 |
| --- | --- | --- | --- | --- | --- | --- |
| 0 | 20.07±2.07 | 18.67±2.91 | 19.35±3.38 | 20±4.25 | 18.25±4.17 | 19.53±4.06 |
| 1 | 19.38±2.05 | 18.9±2.59 | 19.34±2.89 | 20.49±3.7 | 20.18±3.83 | 19.9±3.31 |
| 2 | 18.06±2.89 | 17.55±3.13 | 17.96±3.5 | 18.89±4.07 | 18.58±4.02 | 18.4±3.75 |
| 4 | 16.43±3.28 | 15.76±3.38 | 16.17±3.71 | 17.07±4.05 | 16.69±3.9 | 16.65±3.8 |
| 8 | 16.98±3.87 | 16.61±4.09 | 16.96±4.45 | 18.15±4.67 | 17.73±4.38 | 17.35±4.21 |
| 16 | 17.29±4.83 | 17.05±5.05 | 17.29±5.37 | 18.25±5.46 | 18.05±5.32 | 17.71±5.19 |

# Section 2. Demineralisation results

Demineralisation of tooth powder prior to DNA extraction led to an increase in DNA yield for all samples that had DNA at detectable levels in their non-demineralised extract except in one instance. In five instances, DNA was detected in demineralised samples when the non-demineralised portion had no detectable DNA. Nine of the 12 cementum samples yielded DNA in both fractions with the other three yielding DNA from the demineralised portion only. All coronal dentine samples (11) had no detectable DNA in the non-demineralised portions and very low levels of DNA in two demineralised portions, with the rest having no detectable DNA. Half of the root dentine samples (6) had detectable, although low, levels of DNA in the non-demineralised portions and only four samples had low levels of DNA detected in the demineralised portions. Only data from samples generated using the standard technique (i.e non demineralised samples) was used in the statistical analysis except in construction of the heatmap of STR results where the demineralised sample result were used if a greater number of loci were retrieved. (This was the case for 7 extracts, 6 cementum and 1 root dentine).

# Table C. Average qPCR results for demineralisation vs no demineralisation

| SAMPLE | DECAL WEIGHT | DECAL ng/mg | NON DECAL WEIGHT | NON DECAL ng/mg |
| --- | --- | --- | --- | --- |
| 405A | 70.2 | 0.734797721 | 55.3 | 0.310759494 |
| 405C | 42 | 0.014021429 | 62.4 | 0.005346154 |
| 413A | 32.1 | 0.006903427 | 31.1 | 0.001755627 |
| 413B | 45.2 | 0 | 43.9 | 0 |
| 413C | 23.1 | 0 | 21.7 | 0 |
| 415A | 62.4 | 0.178900641 | 61.5 | 0.056312195 |
| 415B | 34.2 | 0.006298246 | 43.5 | 0 |
| 415C | 53.1 | 0.005954802 | 54.4 | 0.002873162 |
| 418A | 35.3 | 0.009798867 | 25.2 | 0.010440476 |
| 418B | 28.1 | 0 | 30.5 | 0 |
| 418C | 26.2 | 0 | 26.8 | 0 |
| 420A | 31.4 | 0.055541401 | 41.2 | 0.010276699 |
| 420B | 29.2 | 0 | 33 | 0 |
| 420C | 28.4 | 0.016764085 | 31 | 0.014109677 |
| 425A | 46.8 | 0.014876068 | 46.8 | 0 |
| 425B | 41.3 | 0 | 38.2 | 0 |
| 425C | 49.9 | 0 | 46.3 | 0.00387473 |
| 428A | 63.3 | 0.420139021 | 55.5 | 0.041769369 |
| 428B | 38.6 | 0.004430052 | 36.8 | 0 |
| 428C | 35.4 | 0 | 36.8 | 0 |
| 410A | 34.8 | 0.088522989 | 32.9 | 0.002534954 |
| 410B | 56.3 | 0 | 43.8 | 0 |
| 410C | 34.8 | 0.034442529 | 51.5 | 0.011673786 |
| 403A | 70.2 | 0.016794872 | 55.3 | 0 |
| 403B | 44.6 | 0 | 50.2 | 0 |
| 403C | 42 | 0 | 62.4 | 0.002605769 |
| 455A | 30.9 | 0.047404531 | 28.3 | 0 |
| 455B | 38.8 | 0 | 35.2 | 0 |
| 455C | 36.1 | 0 | 39.3 | 0 |
| 474A | 38.4 | 0.336880208 | 41.5 | 0.002819277 |
| 474B | 22 | 0 | 22.2 | 0 |
| 474C | 24.2 | 0 | 22.4 | 0 |
| 4137A | 67.6 | 2.925147929 | 55.9 | 1.093738819 |
| 4137B | 37.3 | 0 | 35.5 | 0 |
| 4137C | 58.4 | 0 | 60.2 | 0 |
|  |  |  |  |  |
| A = cementum | |  |  |  |
| B = crown dentine | |  |  |  |
| C = root dentine | |  |  |  |

# Section 3. Statistical analysis - on the topic of p-values and prediction and confidence intervals for linear mixed effects models

When performing our analyses via linear mixed effect modelling, we employed the package ‘lme4’ in the R-statistical software. The summary function in the lme4 package does not return p-values for each coefficient in the model, and this is because the denominator degrees of freedom for the F-distribution is unknown (although the numerator degrees of freedom is one). This F-distribution is then used to find the t-distribution that becomes the reference distribution for each coefficient (Douglas Bates, personal communication, May 19, 2006)[1].

The issue of having no reference distribution for the coefficients of the linear mixed effects model does not limit our ability to accurately perform model selection. We employ model comparison via ANOVA for two models, where the null hypothesis is “*we have not changed the ability of the model to describe the data”*. If for a parent model $M_{1}$ and a nested model $M_{2}$, we perform ANOVA and return a p-value greater than 0.05, then our interpretation is that $M_{1}$ is not a significantly better model for describing the data than $M_{2}$. Hence we prefer the simpler model $M_{2}$, and discard $M_{1}$. Conversely, for a p-value less than 0.05, our interpretation is that $M_{2}$ is a significantly worse model for describing the data than $M_{1}$, hence we retain $M_{1}$.

Our method is as follows. We start with the full interaction model, and removing terms, we iteratively test whether or not we can simplify the model (starting with highest order interaction terms). When we can no longer simplify the model, we used the final parent model and return the greatest (least significant) p-value obtained when performing ANOVA for the final set of nested models.

Similarly, due to the lack of reference distribution for our coefficients, we cannot derive analytical confidence intervals for the coefficients, or prediction intervals for the model. Approximate prediction intervals can be obtained, but we consider these misleading at best. However, some measure of predictive variability is a desirable trait of any analysis. To this end we produce empirical prediction intervals via 10,000 MCMC samples, and present these for the interested reader.

# S1 Fig Predicted fragment yield for nuclear 67bp fragment at varying PMI, soil temperature and subject age with prediction intervals.

# S2 Fig Predicted fragment yield for nuclear 156bp fragment at varying PMI, soil temperature and subject age with prediction intervals.

# S3 Fig Predicted fragment yield for mitochondrial 77bp fragment at varying PMI and soil temperature with prediction intervals.

[1] https://stat.ethz.ch/pipermail/r-help/2006-May/094765.html
